# Remodeling of Mitochondrial Flashes in Muscular Development and Dystrophy in Zebrafish

**DOI:** 10.1371/journal.pone.0132567

**Published:** 2015-07-17

**Authors:** Meiling Zhang, Tao Sun, Chongshu Jian, Lei Lei, Peidong Han, Quanlong Lv, Ran Yang, Xiaohai Zhou, Jiejia Xu, Yingchun Hu, Yongfan Men, Yanyi Huang, Chuanmao Zhang, Xiaojun Zhu, Xianhua Wang, Heping Cheng, Jing-Wei Xiong

**Affiliations:** 1 Beijing Key Laboratory of Cardiometabolic Molecular Medicine, Institute of Molecular Medicine, Peking University, Beijing, China; 2 State Key Laboratory of Biomembrane and Membrane Biotechnology, Peking University, Beijing, China; 3 The Peking-Tsinghua Center for Life Sciences, Peking University, Beijing, China; 4 College of Life Sciences, Peking University, Beijing, China; 5 Biodynamic Optical Imaging Center, Peking University, Beijing, China; 6 College of Engineering, Peking University, Beijing, China; University of Minnesota, UNITED STATES

## Abstract

Mitochondrial flash (mitoflash) is a highly-conserved, universal, and physiological mitochondrial activity in isolated mitochondria, intact cells, and live organisms. Here we investigated developmental and disease-related remodeling of mitoflash activity in zebrafish skeletal muscles. In transgenic zebrafish expressing the mitoflash reporter cpYFP, *in vivo* imaging revealed that mitoflash frequency and unitary properties underwent multiphasic and muscle type-specific changes, accompanying mitochondrial morphogenesis from 2 to 14 dpf. In particular, short (S)-type mitoflashes predominated in early muscle formation, then S-, transitory (T)- and regular (R)-type mitoflashes coexisted during muscle maturation, followed by a switch to R-type mitoflashes in mature skeletal muscles. In early development of muscular dystrophy, we found accelerated S- to R-type mitoflash transition and reduced mitochondrial NAD(P)H amidst a remarkable cell-to-cell heterogeneity. This study not only unravels a profound functional and morphological remodeling of mitochondria in developing and diseased skeletal muscles, but also underscores mitoflashes as a useful reporter of mitochondrial function in milieu of live animals under physiological and pathophysiological conditions.

## Introduction

The mitochondrion is a dynamic and versatile organelle in virtually all eukaryotes. In addition to serving as the power house of the cell, mitochondria also play a major role in the regulation of intracellular calcium signaling, redox homeostasis, cell cycle control, and apoptotic and necrotic cell death [1]. Emerging functional and proteomic evidence suggests that mitochondria are highly diversified in a tissue- and cell type-specific manner and undergo remarkable remodeling in different developmental stage or in the contexts of stress and disease. For instance, mitochondria undergo remarkable changes during embryonic ventricular cardiomyocytes, displaying from fragmented mitochondria with few cristae and a less-polarized transmembrane potential at embryonic day 9.5 to elongated and interconnected mitochondrial networks at embryonic day 13.5 [2]. Intriguingly, the mitochondrial permeability transition pore and reactive oxygen species (ROS) appear to control cardiac mitochondrial maturation and myocyte differentiation [2]. During myogenic differentiation, the short, fragmented mitochondria of myoblasts develop into elongated, inter-connected mitochondrial network of myotubes, during which mitochondrial differentiation is dependent on nitric oxide inhibition of Drp1-engaged mitochondrial fission [3]. Either cyclophilin D inhibitor cyclosporine A (CsA) or depletion of the gene encoding Cyclophilin D (Ppif) leads to abnormalities of mitochondrial structure and function in skeletal muscles [4, 5]. Similarly, the bioenergetics and fission of mitochondria, which are modulated by the phosphorylation state of Drp1, control calcium homeostasis and hence regulate neuronal development and connectivity [6].

Recently, we have shown that respiring mitochondria undergo intermittent bursts of superoxide and reactive oxygen species (ROS) production, namely “superoxide flashes” which can be visualized by the biosensor mt-cpYFP [7] or the chemical probes mitoSOX and 2, 7-dichlorodihydrofluorescein diacetate [8, 9]. Multi-parametric measurements by us and others have shown that superoxide flashes are a compound phenomenon comprising multifaceted and intertwined processes including ROS bursts, transient dissipation of the mitochondrial membrane potential, mitochondrial permeability transition (MPT) and swelling, and a mitochondrial alkalization (i.e., a “pH flash”) [10–14]. Hence, they are called “mitochondrial flashes” or “mitoflashes” for the entirety of this mitochondrial phenomenon [7, 15, 16]. Owing to its dual sensitivity to superoxide and pH [7], cpYFP measures both superoxide flash and pH flash at once and is therefore a robust mitoflash reporter. Occurring in isolated mitochondria, intact cells, *ex vivo* beating hearts, and live organisms, the mitoflash is a highly-conserved, universal, and physiological mitochondrial activity. The genesis and regulation of mitoflash activity appears to be intimately interwoven with core mitochondrial functions (e.g., energy metabolism) and mitochondrial response to stressors such as exogenous ROS, hypoxia- and anoxia-reoxygenation injury, and apoptotic insults (see [16] for a recent review) as well as epidermal wounding [17]. In adult mammalian skeletal muscle, mitoflashes respond to whole-body glucose- and insulin-mediated metabolic stimulation [11]. In mammalian neuronal progenitor cells, mitoflashes negatively regulate the self-renewal of cortical neural progenitors but positively regulate neuronal differentiation [18, 19]. In worms, the frequency of mitoflash occurrence exhibited multiphasic dependence on age and its level in healthy young and reproductive animals can even predict the overall span of the animal’s adult life [15].

While interest on mitochondrial bioenergetics and signaling permeates the biomedical science, mitoflash imaging could afford a novel optical readout of mitochondrial function in milieu of live animals, at the resolution of a single organelle. Here we tested the hypothesis that mitoflashes may represent a physiological marker of mitochondrial remodeling during the skeletal muscle development in live zebrafish. In addition, we intended to characterize whether and how mitoflash activity alters in skeletal muscle pathology in a zebrafish model of muscular dystrophy. By *in vivo* imaging of mt-cpYFP-expressing transgenic zebrafish, we demonstrated striking multiphasic changes in mitoflash frequency and unitary characteristics during skeletal muscle development (2–14 dpf), alongside mitochondrial morphological remodeling and functional maturation. Investigating mitoflash activity also provided new insights into early involvement of mitochondria in the progression of muscle pathology.

## Materials and Methods

### Zebrafish lines

Wild-type and Tg(*β-actin*:mt-cpYFP) zebrafish were raised and handled in accordance with the animal protocol “IMM-XiongJW-3” for this study, which was approved by the Institutional Animal Care and Use Committee (IACUC) of Peking University that is fully accredited by the AAALAC. The cpYFP cDNA (807 bp) tagged with the COX IV mitochondrial localization signal (COX IV MTS) [7] was cloned and driven by the chicken *β*-actin promoter in the pT2KXIG△in-linker modified vector [20, 21]. The resulting plasmid was injected into one-cell embryos at 75 pg/egg. The transposase messenger RNA (mRNA) was co-injected at 50 pg/egg. The injected embryos were raised to adulthood, from which 5 F_0_ founders were identified by examining mt-cpYFP expression in F_1_ embryos. Heterozygous Tg(*β-actin*:mt-cpYFP)^pku341^ transgenic zebrafish were maintained for the experiments.

### Morpholino injections

MO was targeted to overlap the boundary of zebrafish dmd exon 6 and its downstream intron and induce exon-skipping [22], from exon 5 to exon 7, resulting in a frameshift and premature termination in exon 7 (Gene Tools LLC, Eugene, Oregon, USA), with the sequence 5’-AAAGCGAAAGCACCTGTGGCTGTGG-3’. One-cell embryos were isolated from paired mating of TL or Tg(*β-actin*:mt-cpYFP) transgenic zebrafish, and were injected by using a WPI-Nanoliter 2000 (World Precision Instruments, Shanghai, China).

### Assays for zebrafish motor function and birefringence

Touch-evoked escape response was measured with embryos at 56 hpf using a scale from 0 to 3, scoring 0 for no movement, 1 for a flicker of movement without swimming, 2 for moving away from the probe but with impaired swimming, and 3 for normal swimming. Birefringence was measured with tricaine-anesthetized embryos at 56 hpf. Measurements were carried out by collecting images with a microscope (Nikon AZ-100) mounted with two polarizing filters and a CCD camera (Olympus DP72). Morphant and control embryos were measured at 56 hpf for mitoflash and motor function, as described [23].

### Cyclosporine A (CsA) treatment

CsA stock solution was prepared at 10 mM in DMSO. Embryos were incubated with 1% DMSO in egg water or 100 μM CsA. Morphant and control embryos were incubated with DMSO or 100 μM CsA from 53 to 56 hpf for the measurements of birefringence and mitoflash frequency, or from 50 to 56 hpf for the analysis of motor function, as previously described [24].

### Immunofluorescence staining

Whole-mount immunofluorescence was performed by a standard procedure [25]. Primary antibodies were used at different dilutions: anti-chicken myosin heavy chain (all fast isoforms) mouse monoclonal antibody with 1:10 dilution (F59; DSHB), anti-chicken myosin light chain 1 and 3f mouse monoclonal antibody with 1:10 dilution (F310; DSHB), anti-dystrophin 1:500 (Cat# D8043, MANDRA1; Sigma), anti-DRP1 1:400 (Cat# NB110-55288, Novas). Secondary antibodies were Alexa Fluor 488 goat anti-rabbit and anti-mouse IgG (Invitrogen), Alexa Fluor 555 goat anti-mouse IgG (Invitrogen). All images were acquired by Zeiss 700 confocal microscope (Carl Zeiss Canada Ltd., Toronto, ON).

### *In vitro* transcription of mRNA and microinjection of zebrafish embryos

*Sod2* cDNA were reverse-transcriped from total mRNA of zebrafish embryos, and its sequences were confirmed by Sanger sequencing. The T7 promoter was added to the upstream of the ORF of the two genes. The primers for *sod2* are:

T7-sod2-F: 5’-gatcacTAATACGACTCACTATAGGgcagcgcatgctgtgcagagtcg-3’;

sod2-R: 5’- tatttatttcttggcagcttggaaacgctcg-3’.

The PCR fragment was purified by a kit (TIANGEN, cat# DP204) and was used as DNA template for making capped mRNA with an *in vitro* transcription kit (Ambion, cat#AM1344). The capped mRNA was then tailed with polyA (Ambion, cat#AM1350). The polyA-tailed mRNA was microinjected into 1-cell embryos at 100 ng/μl (2–3 nl/embryo). Control and *sod2*-injected embryos were cultured and collected for mitoflash analysis by a laser-scanning Zeiss 710 confocal microscope (Carl Zeiss Canada Ltd., Toronto, ON).

### Confocal and two-photon excitation microscopy

The Tg(β-actin:mt-cpYFP) transgenic embryos and larvae were used for measuring mitoflashes in vivo. For live imaging, the fish were embedded in 1.5% low-melting-temperature agarose with 0.16 mg/ml tricaine (Sigma) in E3 water, and imaged using a 40X, 1.3NA oil immersion objective at a sampling rate of 1.57 s/frame on Zeiss 710 confocal microscope (Carl Zeiss Canada Ltd., Toronto, ON), and time-lapse images of 100 frames were acquired continuously each time. Dual excitation imaging of mt-cpYFP was achieved by alternating excitation at 405 nm and 488 nm, and collecting emission at >505 nm. Imaging experiments were performed at room temperature (22–26°C). Two-photon imaging of NAD(P)H autofluorescence was done using 40X/1.3 NA oil immersion lens on a LSM710 laser scanning microscope. Two-photon imaging of NAD(P)H used a Coherent Chameleon laser mode-locked to 720 nm (Coherent Inc., Santa Clara, CA). NAD(P)H fluorescence was collected using a non-descanned detector(NDD) equipped with a 455/50nm filter cube. To quantify mitochondrial NAD(P)H fluorescence after image aquiring, the mitochondria regions are defined with iterative threshold algorithm and mean intensity in these regions are calculated. Digital image processing was performed by using the IDL Software Research Systems (Exelis, Boulder, USA) as well as customized programs. With modifications, the Flashsniper that we developed previously [26] was used for mitoflash detection and parametric measurement.

### Transmission electron microscopy (TEM)

Wild-type zebrafish larvae at 2, 5, 8 and 14 dpf were fixed with glutaraldehyde at a final concentration of 2.5% (v/v) in 0.1 M sodium cacodylate buffer. Samples were rinsed and post-fixed with 1.0% OsO4. Then samples were embedded in the Spur resin and sectioned (80–100nm). The sections were stained with uranyl acetate and lead citrate and observed under a transmission electron microscope JEOL 1010 (JEOL Ltd., Japan).

### Statistical analysis

Statistical analysis was performed on relevant data using the Prism GraphPad software package. The nonparametric unpaired *t* test with either Welch’s correction or Mann-Whitney test was applied to determine statistical significance of the differences as noted. Data were reported as median with interquartile range (for scatter plot analysis) and mean ± SEM (for other analysis). The correlation was calculated with Pearson correlation analysis. *P* <0.05 was considered statistically significant.

## Results

### Mitochondrial morphological remodeling entwines with rapid skeletal muscle development

**Fig 1:**
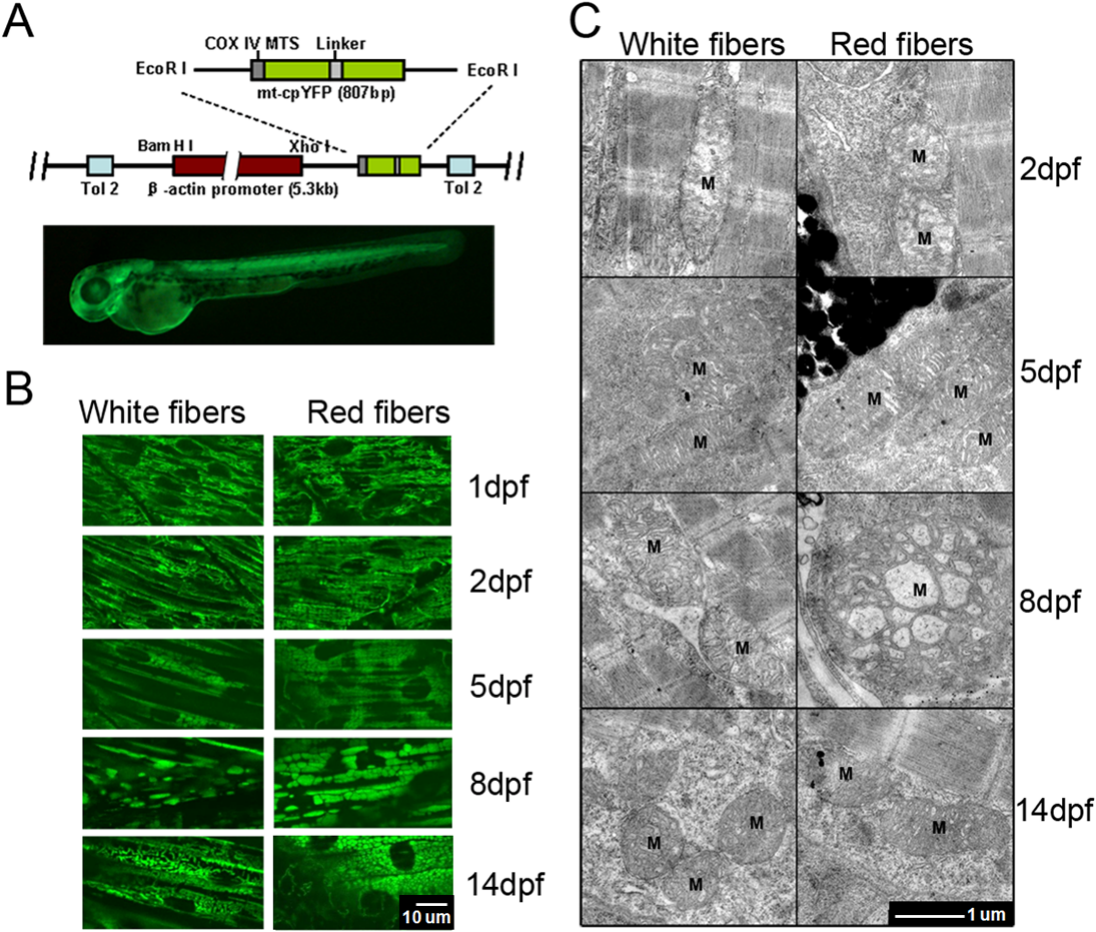
Mitochondrial morphogenesis in developing zebrafish skeletal muscles. (A) Top: A schematic map of Tg(*β-actin*:mt-cpYFP) reporter construct, in which mt-cpYFP was driven by the chicken *β*-actin promoter. The N-terminus of cpYFP was tagged with the COX IV mitochondrial localization signal (COX IV MTS). The transgene was flanked with Tol2 elements (Tol2) to facilitate transgenesis in zebrafish. Bottom: ubiquitous expression of mt-cpYFP in a Tg(*β-actin*:mt-cpYFP) transgenic embryo at 2 dpf. (B) Confocal images of mitochondria in white (left panels) and red (right panels) skeletal muscles of Tg(*β-actin*:mt-cpYFP) embryos from 1 to 14 dpf. Note the fibrillar mitochondria at 1 and 2 dpf, while the rod-like mitochondria at 5, 8 and 14 dpf in white skeletal muscles, whereas large brick-like at 8 dpf and smaller bead-like mitochondria at 5 and 14 dpf in red skeletal muscles. Scale bar, 10 μm. (C) Transmission electron microscopic images of mitochondria (M) in white (left panels) and red (right panels) skeletal muscle cells of Tg(*β-actin*:mt-cpYFP) transgenic embryos from 2 to 14 dpf. Red skeletal muscle cells were identified along with melanocytes. Note fewer immature cristae in mitochondria at 2 dpf but well-developed cristae in mitochondria at 5, 8 and 14 dpf in both red and white muscle fibers. Mitochondria were particularly enlarged in red muscle cells at 8 dpf. Scale bar, 1 μm.

To investigate mitoflash signaling in development and diseases, we generated transgenic Zebrafish with pan-tissue expression of the genetically encoded biosensor cpYFP, using the chicken *β*-actin promoter and the cytochrome C oxidase subunit IV (COX IV) targeting sequence for mitochondrial localization (Fig 1A). By analyzing the levels of cpYFP expression in F_1_ transgenic embryos from 5 individual founders, we found that one transgenic cpYFP line, called as Tg(*β-actin*:mt-cpYFP)^pku341^, had relatively stronger mitochondrial cpYFP expression, which was then used for subsequent experiments in this work. This cpYFP transgenic line had normal growth and reproductivity without apparent abnormality during development and adult stages. In conjunction with *in vivo* confocal imaging, mitochondrial cpYFP expression allowed us to monitor both morphological and functional remodeling of mitochondria in developing skeletal muscles due to the strongest cpYFP signals in this tissue. The red skeletal muscles are located closely to pigmented melenocytes, proximately parallel to the notochord, and are single-nucleus cells. On the other hand, the white skeletal muscles are underneath the red muscles, are arrayed about 45 degree with the notochord, and have 2 or more nuclei. In zebrafish, F310 antibodies are widely used for marking the fast white fibers and F59 antibodies are used for marking the slow red fibers [27, 28]. The two types of skeletal muscle fibers were confirmed either by immunofluorescence staining with F59 and F310 antibodies (S1 Fig).

Morphological changes of mitochondria in both white and red skeletal muscles of intact embryos were tracked from 1 to 14 days post fertilization (dpf) (Fig 1B). However, it was difficult to detect mt-cpYFP signals of embryos beyond 14 dpf due to the thickness of skins. Fig 1B shows rapid and dramatic changes in mitochondrial morphology during skeletal muscle development in zebrafish. The pattern of mitochondrial morphogenesis differed slightly in white and red skeletal muscle fibers. In white muscle fibers, mitochondria showed reticulate and long-tubular appearance at 1 to 2 dpf, fragmented to form banded structure at 5 dpf, and the fragmented mitochondria fused to form elongated tubular mitochondria at 8 dpf, but returned to reticulate and short-tubular appearance at 14dpf. In red muscle fibers, mitochondria had similar morphology from 1 to 5 dpf as that in white muscle fibers, but appeared highly fused and brick-like at 8 dpf and became fragmented and bead-like at 14 dpf. Transmission electron microscopy (TEM) revealed immature mitochondria with fewer cristae at 2 dpf, and more dense cristae in mitochodria at 5 dpf, and large mitochondria with dense cristae at 8 dpf in both white and red skeletal muscle fibers, of which 90% of the mitochondria appeared bigger size and fewer cristae from investigating 10 frames of red fibers and 31 frames of white fibers. Intriguingly, mitochondria at 14 dpf returned to a comparable size as those at 5 dpf in both white and red skeletal muscle fibers (Fig 1C). Since mitochondrial differentiation is dependent on Drp1-engaged mitochondrial fission [3], we also investigated the expression of Drp1 in the skeletal fibers at different stages of zebrafish embryos. Our results showed that Drp1 was gradually decreased from 2 to 8 dpf (S2 Fig), contributing to the enhanced mitochondrial fusion during muscle fiber differentiation. Therefore, both fluorescent mt-cpYFP and TEM images showed that mitochondria undergo dramatic morphological remodeling in muscle fibers during the first 14-day development in zebrafish.

### Robust mitoflash activity in developing zebrafish skeletal muscles

**Fig 2:**
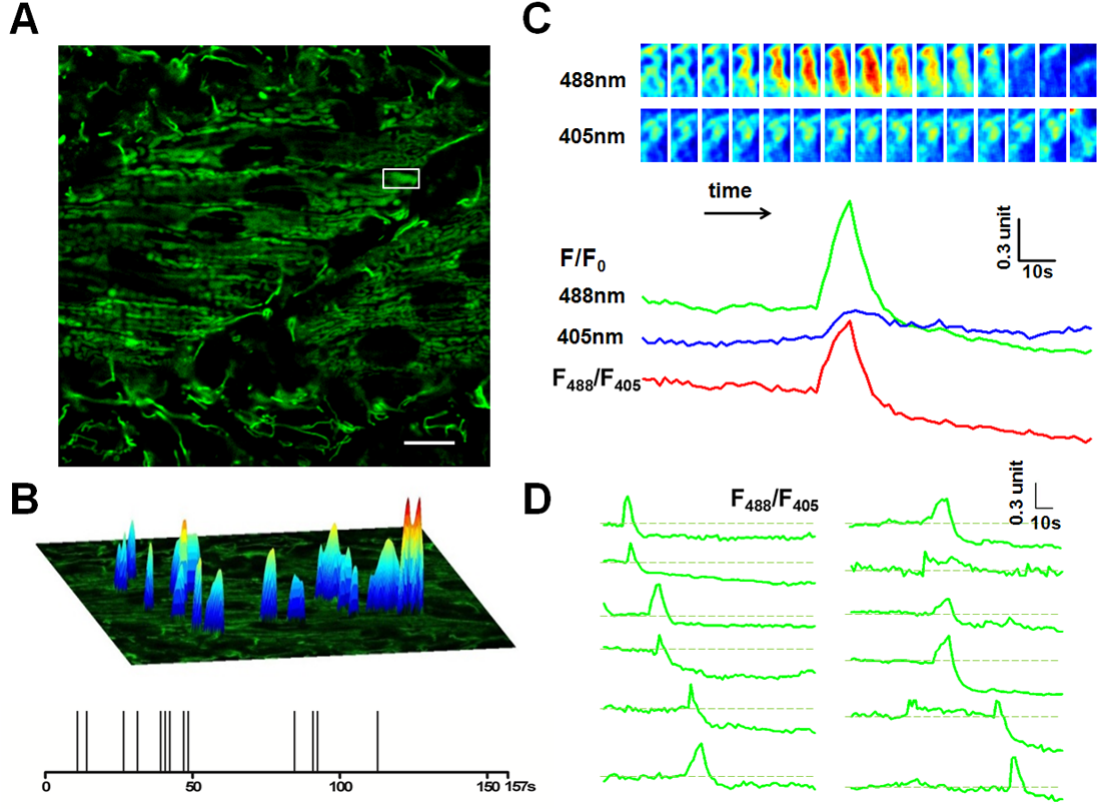
Mitoflash activities in red skeletal muscles of 2 dpf zebrafish embryos. (A). Time-lapse recording of mitoflashes in red skeletal muscles of a mt-cpYFP transgenic embryo. Scale bar, 10 μm. See S1 Movie. (B) Amplitudes, spatial and temporal distribution of the mitoflash signals occurring during a 157-s recording period. The spikes in the temporal diary in the lower panel just reflect the temporal distribution of the mitoflashes, so they are of the same height. The upper surface plots overlaying the mitochondria image mainly indicate the spatial location, intensity and spatial morphology of mitoflashes. Their height is defined according to respective mitoflash peak amplitude ∆F/F_0_, instead of the background intensity. (C) Time course of a representative mitoflash from the boxed region in panel A. Upper panel: time sequence of the mitoflash seen alternatively at 488 nm and 405 nm excitation. Lower panel: line plots of normalized cpYFP signals (F/F_0_) under 488 nm and 405 nm excitation and their ratio (F_488_/F_405_), the latter removes motion artifacts associated with mitochondrial swelling. (D) Diverse morphology of mitoflashes of the same skeletal muscle. The traces were arranged by the start points of the major mitoflash event seen in a single trace. Photobleaching (~10%) was corrected based on global intensity decay and the dashed lines mark the basal level. Traces of the last 31 s are not shown due to motion artifacts caused by muscle twitch (see S1 Movie).

Next, we examined the characteristics of mitoflashes during mitochondrial morphogenesis of skeletal muscles. We observed robust mitoflash activity in red skeletal muscles of embryos at 2 dpf (Fig 2; S1 Movie). Time-lapse *in vivo* imaging revealed that, the number of mitofalshes registered in 100 consecutive images (total 157 s) varied from 0 to 26, with the average value of 4.9 (n = 43). In one of the most active zebrafishes, spontaneous mitoflashes occurred randomly in space and time, with variable intensity, spatial area and morphology (Fig 2B). Fig 2C shows that, as reported by mt-cpYFP fluorescence at 488 nm excitation, a mitoflash rose suddenly and reached its peak of 1.7 units of F/Fo in 11s, decayed precipitously with a half decay time (T50) of 3.4s, and followed by a long undershoot which did not recover until the end of the 157-s recording (Fig 2C). It is noteworthy that mitoflashes were sometimes accompanied by discernible mitochondrial swelling that masqueraded as “mitochondrial contraction” (Fig 2C, S1 Movie) and such a motion artifact can be largely removed by normalizing the mitoflash signal with the fluorescent signal seen with the isobestic excitation of mt-cpYFP at 405 nm, yielding the ratiometric signal F_488_/F_405_ (Fig 2C). Examination of a panel of events from the same skeletal muscle revealed that the vast majority of events comprised a sudden rise, a brisk decay, and a subsequent enduring undershoot, regardless their peak intensity and spatial size (Fig 2D). Furthermore, overexpression of *sod2* mRNA reduced cpYFP fluorescence signals and mitoflash frequency in zebrafish embryos, consistent with the ROS contribution to cpYFP signals (S3 Fig). Thus, mitochondria in live embryonic and larval zebrafish undergo mitoflash activity, supporting the notion that mitoflash reflects a physiological function of the mitochondria. However, temporal characteristics of mitoflashes of skeletal muscles at 2 dpf differed from those of mitoflashes reported previously. Previous studies have shown that a mitoflash exhibits a sudden rise followed by a gradual decay with T50 of 10–20 s on most occasions, including those in skeletal muscles in adult live mice [7] as well as those in the muscle of pharynx of C. elegans [15]. In contrast to these regular mitoflashes, mitoflash activity in zebrafish red skeletal muscles at early development was dominated by short events, the 50% decay time after the peak (T50) lasting merely 3.2 s on average.

### Developmental changes of mitoflash activity in zebrafish skeletal muscles

**Fig 3:**
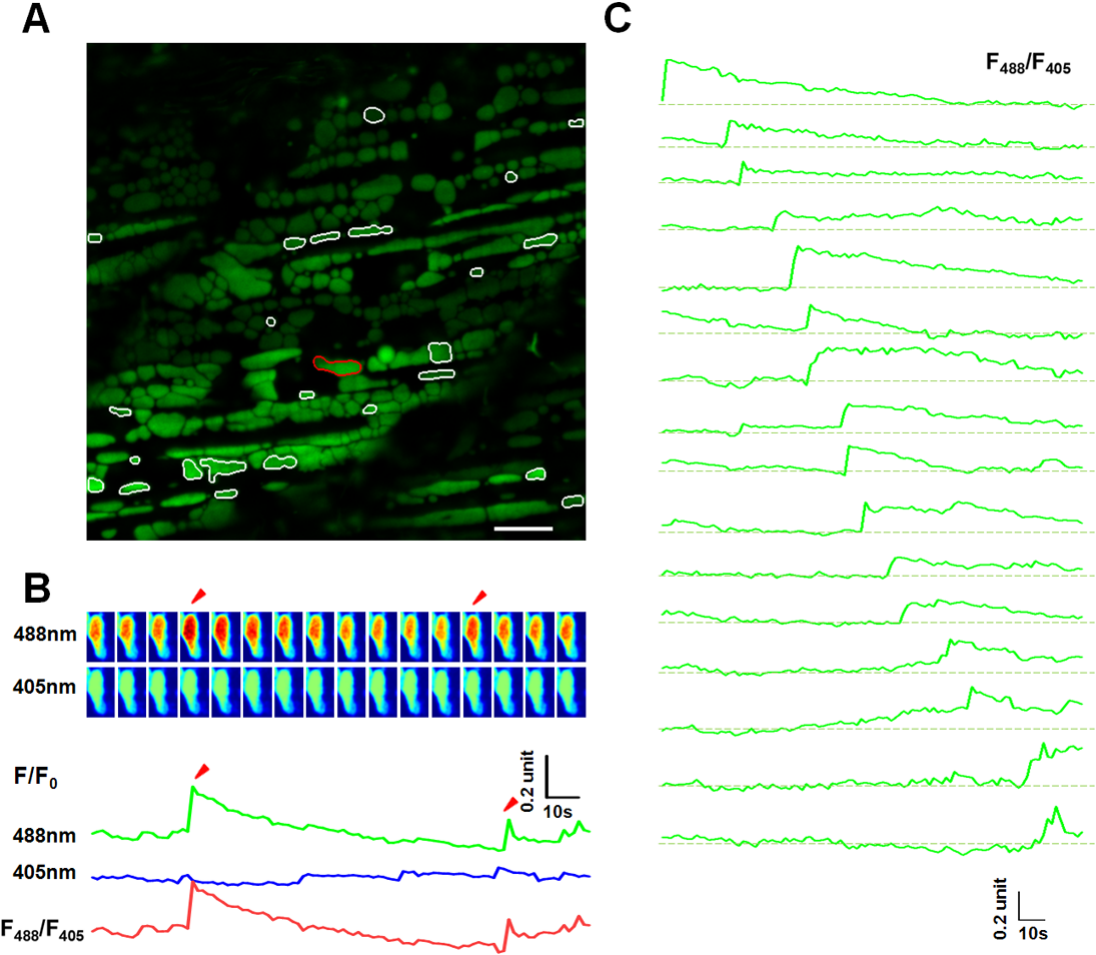
Mitoflash activities in red skeletal muscles of 8 dpf zebrafish embryos. (A) The same as in Fig 2A, except that the data were from a red skeletal muscle at 8 dpf. White contours mark mitochondria undergoing mitoflashes. Scale bar, 10 μm. See also S2 Movie. (B) A representative mitoflash (marked in panel A with red-color coded contour). The same as in in Fig 2B. Arrow heads mark two consecutive mitoflashes in the same mitochondria. Upper panel: time sequence of the mitoflash seen alternatively at 488 nm and 405 nm excitation and their ratio (F_488_/F_405_). Note that the ratiometric line plot, F_488_/F_405_, largely eliminated motion artifacts. (C) A family of traces showing diversified time courses of mitoflashes from red skeletal muscle at this developmental stage. The traces were arranged by the start points of mitoflash events. Photobleaching (~10%) was corrected based on global intensity decay and the dashed lines mark the basal level. Note the distinct developmental differences compared to mitoflashes in Fig 2D.

**Fig 4:**
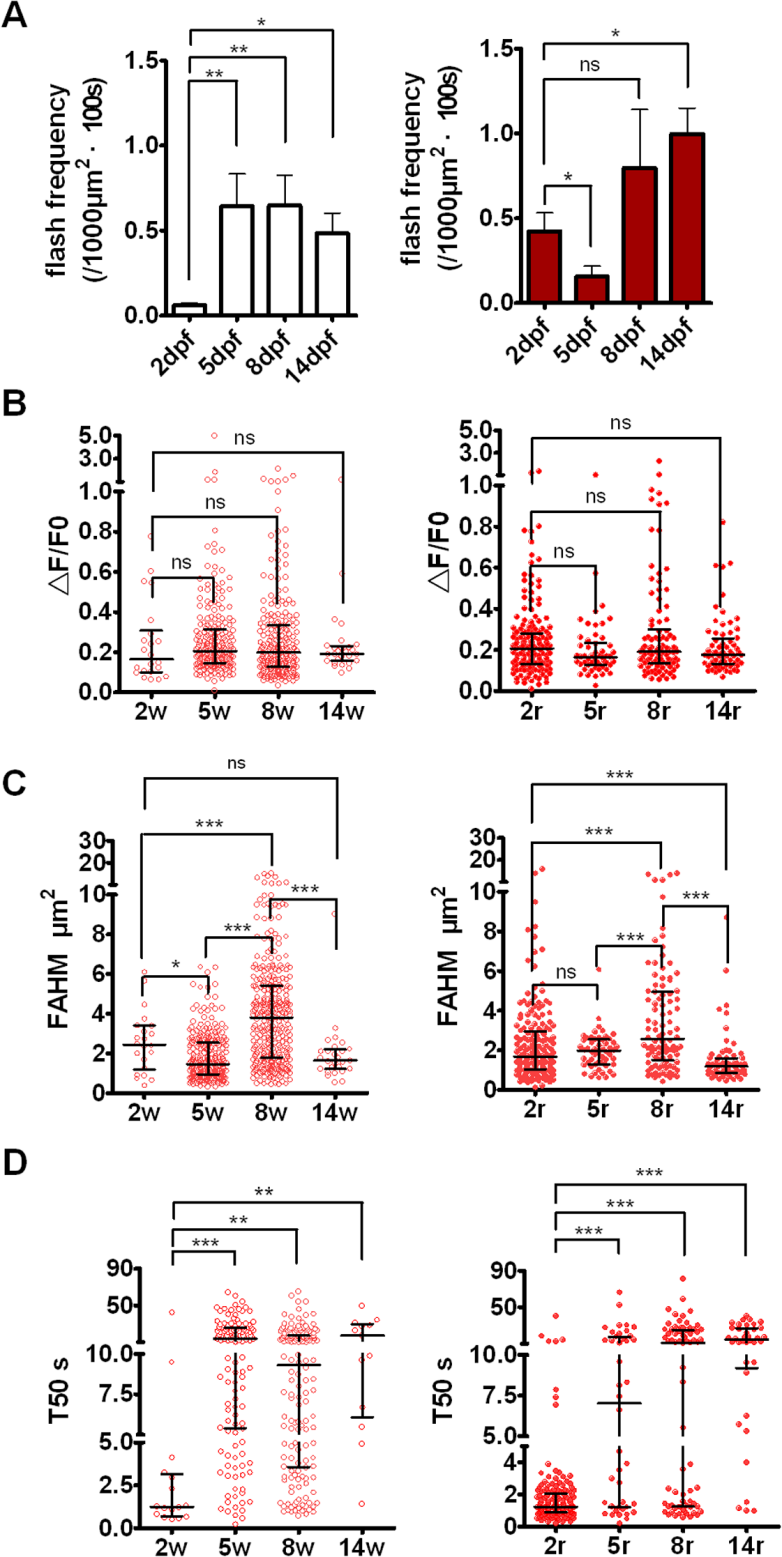
Developmental changes of mitoflashes in skeletal muscle cells. (A) Mitoflash frequency in red (right) and white (left) skeletal muscle cells of cpYFP transgenic embryos at 2, 5, 8 and 14 dpf. (B-D) ΔF/F_0_ amplitude (B), FAHM (C), and T50 (D) of red (right) and white (left) skeletal muscle cells of transgenic embryos at 2, 5, 8 and 14 dpf. For ΔF/F_0_, FAHM, and T50, data are shown as scatter plots to illustrate both scatterness and clusteration. Vertical scale may have a break to cover the data range, and horizontal bars show quartiles at 1/4, 1/2 and 3/4 of the data, respectively. Note that ΔF/F_0_ amplitude appeared to be unchanged during early development, while FAHM and T50 underwent drastic and multiphasic changes. S-type mitoflashes (T50<5s) occurred dominantly in immature skeletal muscles, and R-type mitoflashes (T50 >10s) dominated at 14 dpf, and transitory type of mitoflashes (T50 between 5 and 10s) were also rather frequent at 5 and 8 dpf in white, but not red, skeletal muscles. For mitoflash analysis at 2, 5, 8 and 14 dpf, we examined 7, 4, 4, and 2 embryos, respectively, as well as generated 43, 31, 10 and 6 frames for red muscle fibers and 37, 32, 31 and 5 frames for white muscle fibers, respectively. w, white skeletal muscles; r, red skeletal muscles; 2w, white skeletal muscles at 2 dpf; 2r, red skeletal muscles at 2 dpf. Data were reported as median with interquartile range (for Fig 4B-D) and mean ± SEM (for Fig 4A). The nonparametric unpaired t test with Welch’s correction (for Fig 4B-D) and Mann-Whitney test (for Fig 4A) was applied to determine statistical significance of the differences as noted. For red fibers, the percentages of S-type mitoflashes of embryos at 2, 5, 8 and 14 dpf are 95.2%, 47.6%, 43.7% and 13.5% respectively, while the percentages of R-type mitoflashes of embryos at 2, 5, 8 and 14 dpf are 7.5%, 40.5%, 52.1% and 70.3% respectively. For white fibers, the percentages of S-type mitoflashes of embryos at 2, 5, 8 and 14 dpf are 87.5%, 22.6%, 32.9% and 15.4% respectively, while the percentages of R-type mitoflashes of embryos at 2, 5, 8 and 14 dpf are 12.5%, 57.9%, 46.2% and 53.8%, respectively.

To determine whether the distinct temporal properties of mitoflashes are attributable to a developmental change, we recorded mitoflashes at 2, 5, 8 and 14 dpf. From 5 dpf on, most mitoflashes fell into two distinct categories, one with T50 > 10s and the other with T50 <5s (Figs 3 and 4D). We called them regular (R-type, T50>10s) and short (S-type, T50<5s) mitoflashes. Analysis of ΔF/F_0_ versus T50 showed significant correlation in S-type and R-type mitoflashes (Pearson correlation coefficient = 0.4053 and 0.4039 respectively), but not in transitory (T-type) mitoflashes (5s<T50<10s) (Pearson coefficient = -0.1488) (S4 Fig); no significant correlation was found between mitoflash amplitude and area (FAHM) (not shown), suggesting that mitoflash intensity is relatively independent of mitochondrial size.

Examination of a panel of events from the same red skeletal muscle revealed that the majority of events comprised a sudden rise and a gradual decay but a disappearance of the aforementioned sustained undershoot at 8 dpf (Fig 3C, S2 Movie), which is in contrast to that of mitoflashes at 2 dpf (Fig 2C and 2D). Notably, there were large, brick-shaped mitoflashes at 8 dpf (Fig 3), drastically differing from those at 2 dpf (Fig 2), supporting the notion that changes in the temporal and spatial properties of mitoflashes are tightly associated with mitochondrial morphogenesis during skeletal muscle development in zebrafish.

Simultaneous measurement of mitoflashes in white skeletal muscles revealed that mitoflash activity was scarce in white muscles at 2 dpf (Fig 4A), with the red/white mitoflash frequency ratio reaching 7.0. At 5 dpf, the mitoflash activity was markedly increased in white skeletal muscle, but decreased in red skeletal muscle, reversing the red/white mitoflash frequency ratio to 0.24. Later on, mitoflashes activity in both red and white skeletal muscles reached plateau, with the red/white mitoflash frequency ratio of 1.23 and 2.06 at 8 and 14 dpf, respectively (Fig 4A).

We then analyzed other characteristic parameters of mitoflashes, including the average fractional peak increase (ΔF/F0), full area at half maximum (FAHM), and T50 [7]. Interestingly, we found that the ∆F/F_0_ of mitoflashes was similar from 2 to 14 dpf (Fig 4B), while the FAHM was the highest at 8dpf (Fig 4C), which is consistent with the enlarged size of mitochondria revealed by both fluorescent confocal microscopy (with cpYFP as mitochondrial marker) and transmission electron microscopy at 8 dpf (Fig 1B and 1C). As for the temporal characteristics, mitoflashes were predominantly the S-type in immature red and white skeletal muscles (eg. 2 dpf), but the R-type in matured muscles (14 dpf). At 5 and 8 dpf, three-types of mitoflashes coexisted in white skeletal muscle, whereas the events in red skeletal muscles were more clearly bi-segregated into the S- and R-types (Fig 4D). In addition, there was no significant difference of ∆F/F_0_, FAHM, and T50 between red and white skeletal muscles (Fig 4). Together, these data suggest that mitoflashes display dramatic developmental changes with moderate muscle-type specificity in skeletal muscles of embryonic and larval zebrafish.

### Altered mitoflash activity in skeletal muscles of dystrophin morphants

**Fig 5:**
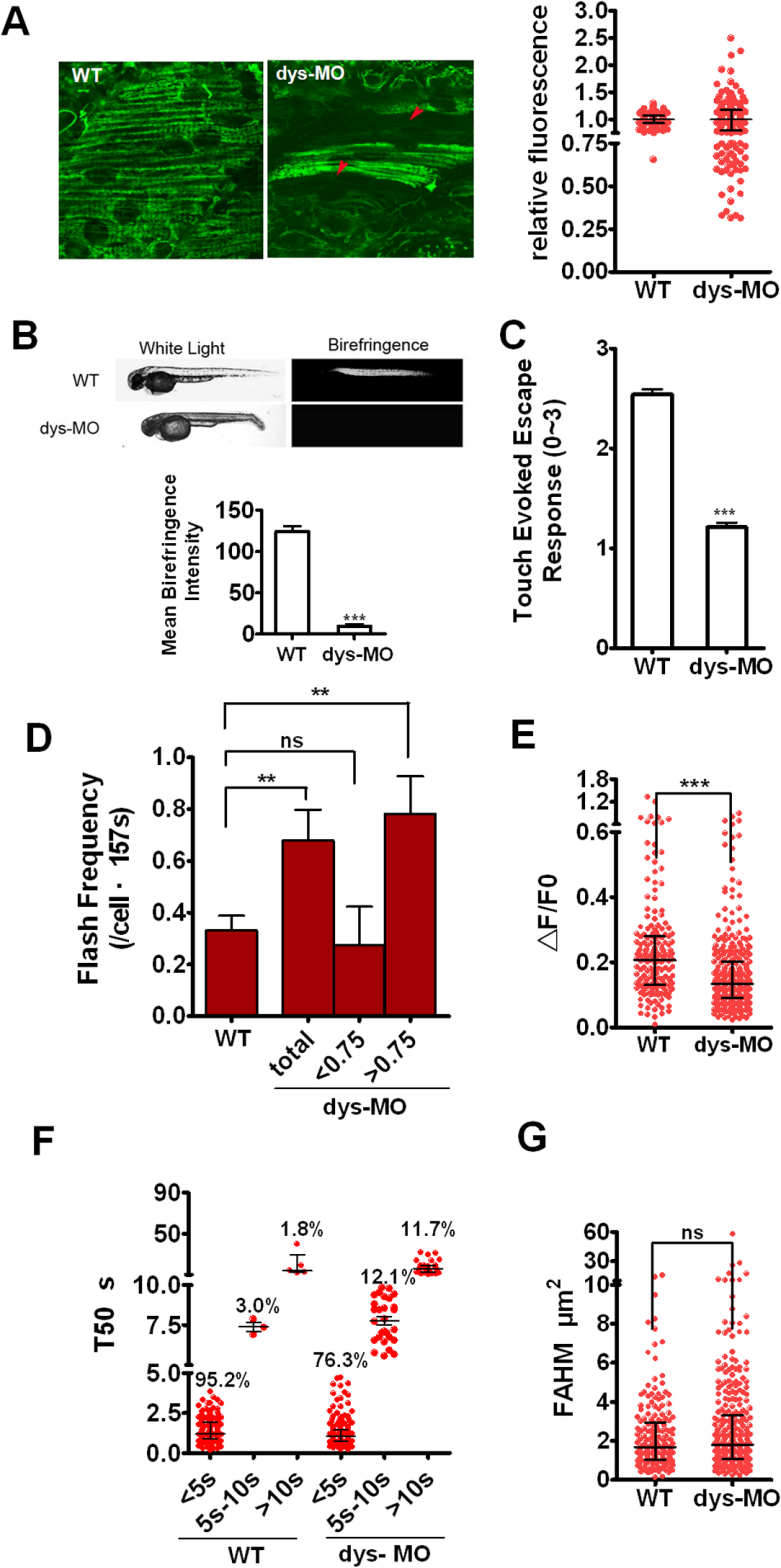
Mitoflashes are switched to lower ΔF/F_0_ amplitude and R-type mitoflashes in dystrophin morphants. (A) Live images of mitoflashes in mitochondria of a wild-type mt-cpYFP transgenic embryo and dys-MO morphant (left) and the relative fluorescence between them (right). Note the heterogeneity of cpYFP signals in red muscle cells in dystrophin morphants, giving rise to a highly scattered cellular cpYFP intensity distribution (A, arrowheads). About half of the cells in morphants showed relative fluorescent <0.75 to the average. (B) Upper: Birefringence was determined in embryos of wild type (WT) and dystrophin morphant (dys-MO). Corresponding bright-field images were shown in the left panels. Note the diminished birefringence in a dys-MO morphant. Lower: statistic of the mean birefringence intensity between the two groups. (C) Touch-evoked escape response was determined in 56 hpf embryos. Note reduced motor function of dys-MO morphants. (D-G) Mitoflash frequency, ΔF/F_0_, T50 and FAHM of red skeletal muscle cells were measured in a wild-type transgenic embryo (WT) and dys-MO morphant (dys-MO). When dys-MO cells were divided into 2 subsets of relative fluorescence, the cells with relative fluorescence >0.75 showed higher frequency that led to overall higher mitoflash frequency in dys-MO morphants than WT. Note ΔF/F_0_ amplitude decreased while FAHM had no changes, but T50 (>10s) increased due to longer T50 for S-type mitoflashes and the emergence of transitory and R-type mitoflashes in dystrophin morphants. For mitoflash analysis of WT embryos and dys-MO morphants at 2 dpf, we examined 8 and 11 individual embryos, as well as generated 43 and 57 frames for red muscle fibers, respectively. Data were reported as median with interquartile range at 1/4, 1/2 and 3/4 of the data, respectively (Fig 5A, E-G) and mean ± SEM (for Fig 5B-D). Unpaired t test with Welch’s correction (Fig 5A-D) and Mann-Whitney test (Fig 5E and G) was applied to determine statistical significance of the differences. **: p <0.01 versus wild-type group, ***: p <0.0001 versus wild-type group.

We next explored possible disease-related changes of mitoflashes by using dystrophin mutants. An ethylnitrosourea-induced genetic dystrophin mutant *sapje* has been shown to recapitulate a spectrum of phenotype of human Duchenne muscular dystrophy (DMD) [22]. We first confirmed the knockdown efficiency of dys-MO (S5 Fig) and dystrophin morphant phenotypes (Fig 5B and 5C), characterized by decreased birefringence, an indicator of muscle fiber disorganization (Fig 5B) and impaired touch-evoked escape, an index of motor function (Fig 5C) compared with those in wild-type controls. With the transgenic expression of mt-cpYFP mitoflash reporter, we then examined whether the characteristics of mitoflashes were affected in dystrophin morphants. We mainly focused on investigating mitoflashes in red skeletal muscles of DMD zebrafish because of their higher mitoflash frequency at 2 dpf for the ease of mitoflash detection and quantification. Unexpectedly, basal mt-cpYFP signal displayed high heterogeneity in red fibers of dystrophin morphants, with significant portions of myocytes displaying elevated or markedly diminished basal fluorescence levels (Fig 5A). Mitoflashes were hardly detected in red muscle cells that displayed low mt-cpYFP signal, probably reflecting an impaired mitochondrial function in apoptotic cells in dystrophin mutants, as previously reported in zebrafish and mice [4, 24, 29]. Interestingly, the mitoflash frequency, which was averaged over cells with clear cpYFP signals, increased; in particular, the mitochondria with relatively higher fluorescence (relative fluorescence >0.75) showed higher mitoflash frequency (Fig 5D). Both R- and T-type mitoflashes increased, but more so for R-type mitoflashes (from 1.8% in wild-type embryos to 11.7% in morphants) in morphants (Fig 5F). While ∆F/F_0_ was decreased (nonparametric Mann-Whitney test, P<0.0001), FAHM was unchanged in dystrophin morphants compared those in wild-type controls (Fig 5E and 5G). These data suggest that there are more degenerated mitochondria and increased R-type mitoflashes in dystrophin morphants.

**Fig 6:**
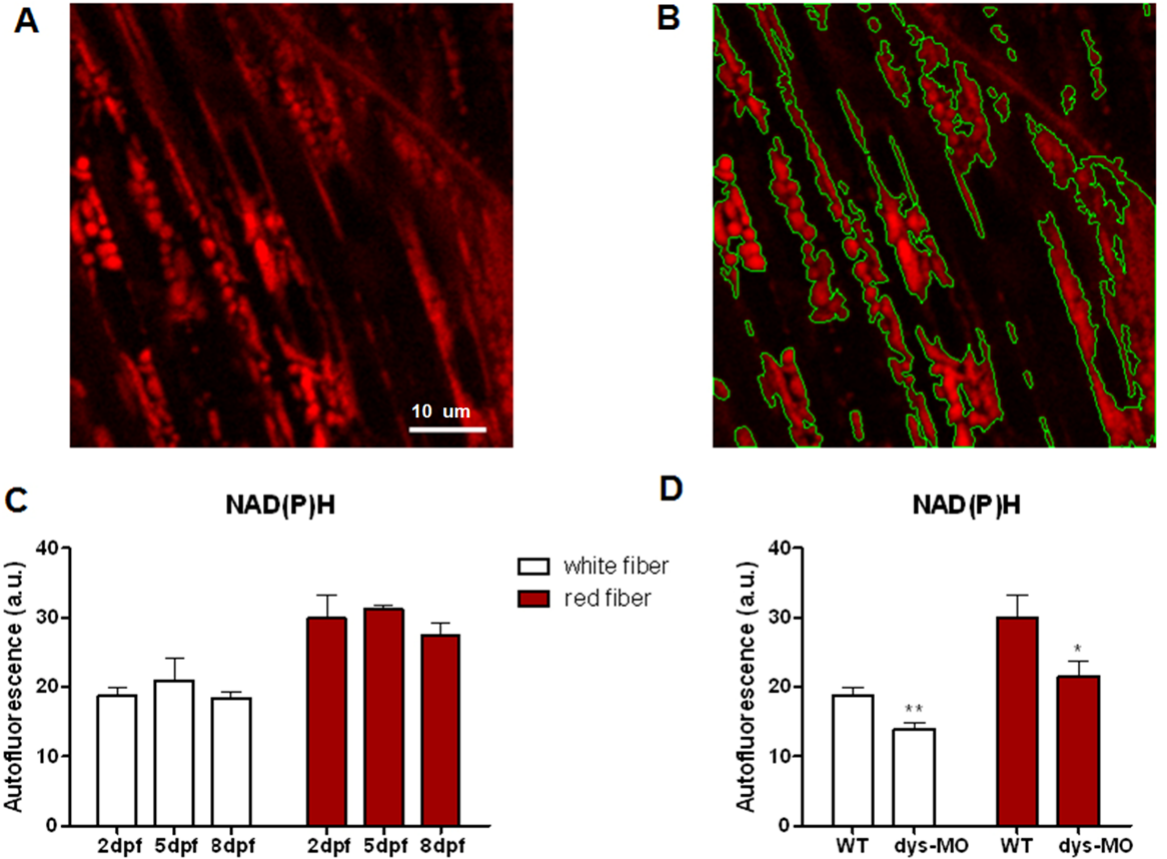
NAD(P)H signal is relatively stable during normal development but reduced in dystrophin morphants. (A) NAD(P)H signal of white skeletal muscle cells of zebrafish embryo at 5dpf. Scale bar,10μm. (B) Intensity-based segmentation of mitochondria. NAD(P)H signal was defined as mean intensity of mitochondrial regions. (C) NAD(P)H autofluorescence in the mitochondria of red (right) and white (left) skeletal muscle cells of wild-type embryos at 2, 5 and 8 dpf. No significant change of NAD(P)H was observed during development in either red or white fibers. N = 5–13 animals per group. (D) NAD(P)H signal was significantly decreased in both red and white fibers of dys-MO morphants (dys-MO) compared with wild-type (WT) controls. Data are expressed as the mean±SEM. *: p<0.05; **: p<0.01 versus wild-type group.

To further examine possible role of mitoflashes in muscular disease, we first imaged autofluorescence of NAD(P)H, which level reflects the mitochondrial respiration chain activity. We found that that NAD(P)H was significantly reduced in dystrophin morphants in both red and white skeletal fibers (Fig 6), consistent with substantially compromised mitochondrial function in dystrophin morphants. Meanwhile, we also found that, despite dramatic mitochondrial morphological and mitoflash changes during development, NAD(P)H intensity exhibited little or no changes, suggesting that remodeling is caused by a mechanism downstream of basic metabolism during normal early development. Next, we tested effects of CsA, an inhibitor of Cyp-D, on dystrophin morphant phenotypes and mitoflash activity. Remarkably, CsA rescued the touch-evoked escape response (motor function) and birefringence (muscle fiber organization) in dystrophin morphants (S6B and S6C Fig). Concomitantly, CsA markedly decreased both R- and T-type mitoflash frequency in dystrophin morphants (S6A and S6F Fig). In contrast, CsA exerted little effect on mitoflash frequency in wild-type zebrafish, consistent with previous reports [9, 30]. However, CsA failed to restore the ∆F/F_0_ and FAHM in dystrophin morphants (S6D and S6E Fig). Taken together, these data reveal an intimate relationship between mitoflash activity and pathology of Duchenne muscular dystrophy.

## Discussion

Mitochondria are well-perceived to have important functions in regulating organ development, and are central to a broad spectrum of pathologies. By transgenic pan-tissue expression of the genetically encoded fluorescent mitoflash reporter cpYFP, in conjunction with *in vivo* confocal microscopy, here we provided a novel means for investigation of mitochondrial morphological remodeling and functional activity in developing skeletal muscles of zebrafish, and explored possible early mitochondrial alterations in a zebrafish model of DMD. Our major findings are as the following.

First, we have identified distinct types of mitoflashes, the S-type and the R-type, alongside a transitory type of mitoflashes (T-type), on the basis of their kinetics of decay. Moreover, we have uncovered, for the first time, dramatic changes of mitoflash characteristics in a developmental program. Particularly, in both red and white skeletal muscles, the S-type mitoflashes dominated at 2dpf, but were progressively switched to the R-type during development, such that the R-type mitoflashes dominated at 14 dpf. The switch of mitoflash types aligns well with mitochondrial morphological changes, suggesting that it presents a physiological marker for mitochondrial functional maturation.

Second, we have shown that the rate of mitoflash occurrence exhibited multiphasic, muscle-type specific changes during development. In red skeletal muscles, mitoflash activity was turned on 2 dpf but underwent a significant decrease at 5 dpf. In contrast, it was extremely low at 2dpf but quickly reached its plateau level at 5 dpf during white skeletal muscle development. The much higher mitoflash frequency in red muscle cells compared with that in white muscle cells at 2 dpf is consistent with that red muscle cells develop earlier than white muscle cells [31]. These data further support the notion that mitoflashes act as a physiological reporter of mitochondrial function in live animals.

Furthermore, we have shown that the functional remodeling of mitochondria in developing skeletal muscles was associated with equally dramatic, multiphasic changes in mitochondrial morphology. In white and red skeletal muscles, early mitochondria of tubule shape interconnected to form reticulate network at 1–2 dpf. Later, they fragmented, fused, and reorganized in a muscle-type specific manner, with thick tubular mitochondria formed in white skeletal muscles and large brick-like mitochondria appeared in red skeletal muscles at 8 dpf. Eventually, white skeletal muscle mitochondria consisted of thin tubular mitochondrial network, whereas red skeletal muscles were packed with bead-like mitochondria. Parallel ultrastructure study affirmed the developmental change of mitochondrial size and extended this finding by showing gradual maturation of mitochondrial cristae during skeletal muscle development. The bigger size and fewer cristae of mitochondria at 8 dpf might reflect a change of metabolic status when the embryos at this stage switches the way of gaining nutrition, a transition at intake of nutrition from the yolk to food. Future studies need to address the underlying mechanisms of mitochondrial morphogenesis.

However, we do not have a full understanding of the mitoflash undershoot frequently seen in early development, but disappeared when muscles mature. One hypothesis is that it reflects a long-lasting acidification of the mitochondria after the initial phase of a mitoflash, because similarly sustained acidification signals have been recently found in neurons [32]. If this was the case, mitochondria in early development should be prone to proton-leak as compared to those in matured skeletal muscles. In addition, since we failed to detect any developmental change in the NAD(P)H level, possible mechanisms underlying mitochondrial remodeling likely reside downstream of NAD(P)H metabolism.

Mitochondrial dysfunction and apoptosis are considered as the major cellular mechanisms in muscular dystrophy of mice [4, 29, 33, 34]. Mitochondria in muscular dystrophy are highly permeable to protons that are unfavorable for the build-up of transmembrane proton gradients. This leads to proposing that excessive mitochondrial reactive oxygen species (ROS) are produced in muscular dystrophy [35]. In a dystrophin mutant, a zebrafish model for DMD, we first report that mitoflashes in red muscle cells had reduced ΔF/F_0_ amplitude and increased mitoflash frequency and R-type mitoflashes while motor function and birefringence were reduced at 2dpf. Using two-photon microscopic measurement of NADH autofluorescence, we have also provided a link between pathological mitoflashes to that of mitochondrial respiration in dystrophin morphants.

In summary, we have demonstrated a profound developmental remodeling of mitoflash activity that accompanies with striking developmental changes in mitochondrial morphogenesis. Apart from muscle-specific and multiphasic changes in mitoflash frequency, mitofash remodeling includes the presence of S-type mitoflashes in early embryos, the coexistence of S-, T- and R-type mitoflashes during maturation, and the switch to R-type mitoflashes in mature skeletal muscles. In addition, we have documented mitoflash changes in early development of muscular dystrophy, including remarkable cell-to-cell heterogeneity with increased mitoflash frequency, altered S- to R-type mitoflash transition and reduced NAD(P)H. This study not only underscores an important role of mitochondria in skeletal muscle development and diseases, but also illustrates the usefulness of mitoflashes as an *in vivo* reporter of mitochondrial function in physiological and pathophysiological conditions.

## Supporting Information

S1 Fig Immunostaining distinguished the red and white fibers of skeletal muscles. (DOC)

S2 Fig Drp1 were decreasing during zebrafish skeletal muscle development. (DOC)

S3 Fig Basal cpYFP signal was decreased in zebrafish embryos overexpressing *sod2*. (DOC)

S4 Fig Pearson correlation analysis of ΔF/F_0_ and T50 yielded coefficients of 0.4053 for S-type, -0.1488 for T-type, and 0.4039 for R-type mitoflashes. (DOC)

S5 Fig Dystrophin was efficiently reduced by dys-MO. (DOC)

S6 Fig CsA partially rescued dysphophin morphant phenotype and decreased mitoflash frequency in red fibers of dystrophin morphants at 2 dpf. (DOC)

S1 Movie Mitoflash activities in a red skeletal muscle at 2 dpf. (AVI)

S2 Movie Mitoflash activities in a red skeletal muscle at 8 dpf. (AVI)
