# Supplementary material for: Remodeling of Mitochondrial Flashes in Muscular Development and Dystrophy in Zebrafish
Source: PLoS One. 2015 Jul 17;10(7):e0132567. doi: 10.1371/journal.pone.0132567 (PMC4506073; doi:10.1371/journal.pone.0132567)
Supplement: S1 Fig — (DOC) [file pone.0132567.s001.doc]

**
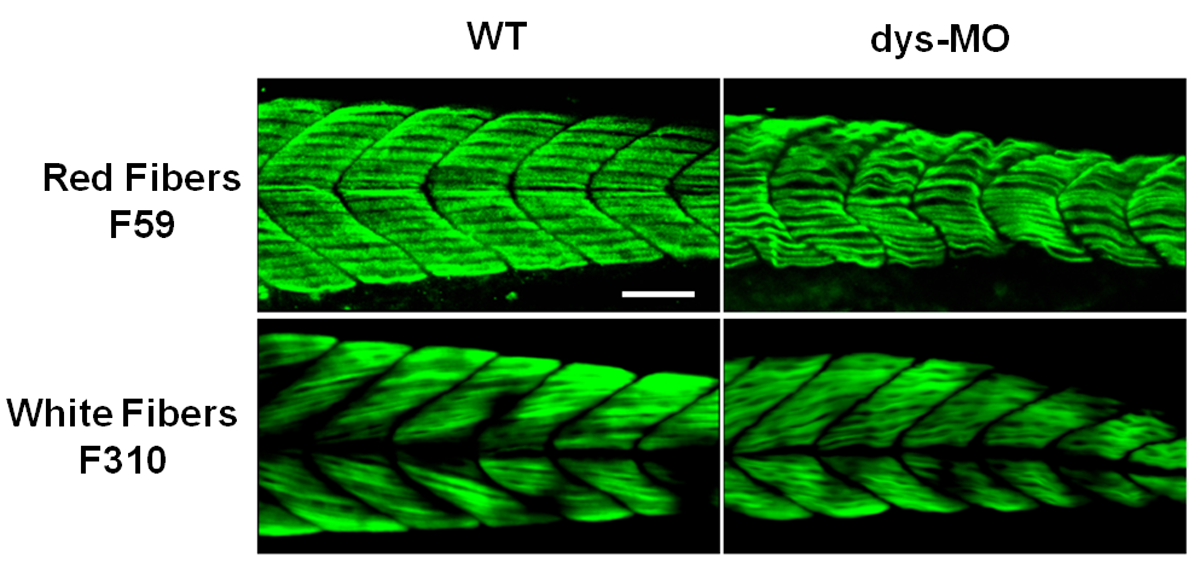
**

**S1 Fig. Immunostaining distinguished the red and white fibers of skeletal muscles.** Red fibers were labeled by F59 antibody for slow myosin (n=20) and white fibers were labeled by F310 antibody for fast myosin (n=18). Distinctive mitochondrial morphologies were found in these fiber types at 2 dpf as shown in Figure B and S1 Movie. Note abnormalities of these fibers in dys-MO-injected morphants. Scale bar, 50 µm.
