# Supplementary material for: Remodeling of Mitochondrial Flashes in Muscular Development and Dystrophy in Zebrafish
Source: PLoS One. 2015 Jul 17;10(7):e0132567. doi: 10.1371/journal.pone.0132567 (PMC4506073; doi:10.1371/journal.pone.0132567)
Supplement: S2 Fig — (DOC) [file pone.0132567.s002.doc]

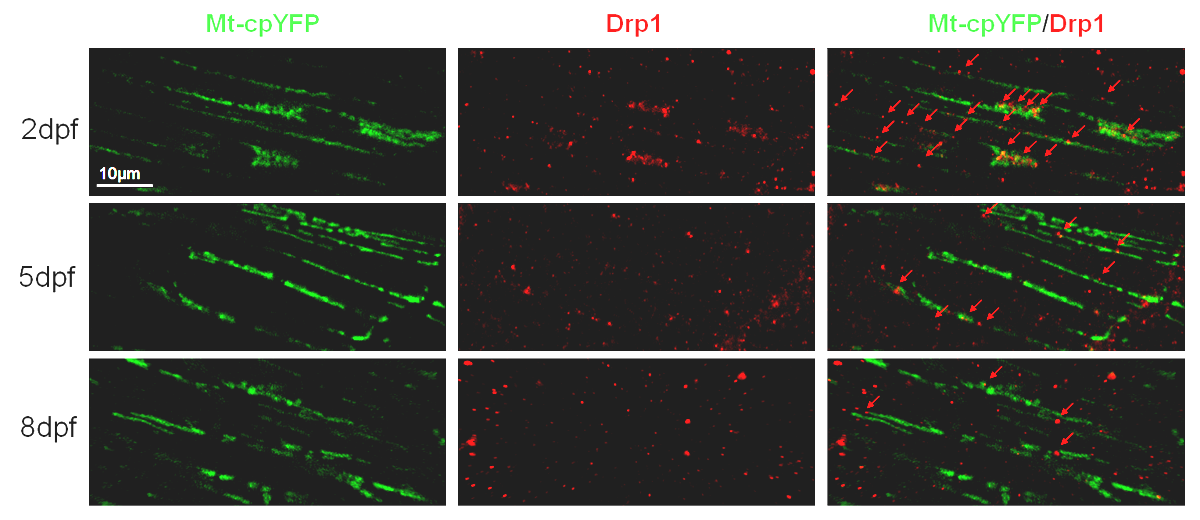


**S2 Fig. Drp1 were decreasing during zebrafish skeletal muscle development.** Note that Drp1 was recruited overlapped with or between Tg(*β*-actin:mt-cpYFP)-positive mitochondria, decreasing from 2-dpf (n=18), 5-dpf (n=15) to 8-dpf embryos (n=14). Due to severe cpYFP signal loss after fixation, we only investigated mt-cpYFP signals in white fibers.
