# Supplementary material for: Remodeling of Mitochondrial Flashes in Muscular Development and Dystrophy in Zebrafish
Source: PLoS One. 2015 Jul 17;10(7):e0132567. doi: 10.1371/journal.pone.0132567 (PMC4506073; doi:10.1371/journal.pone.0132567)
Supplement: S3 Fig — (DOC) [file pone.0132567.s003.doc]

**
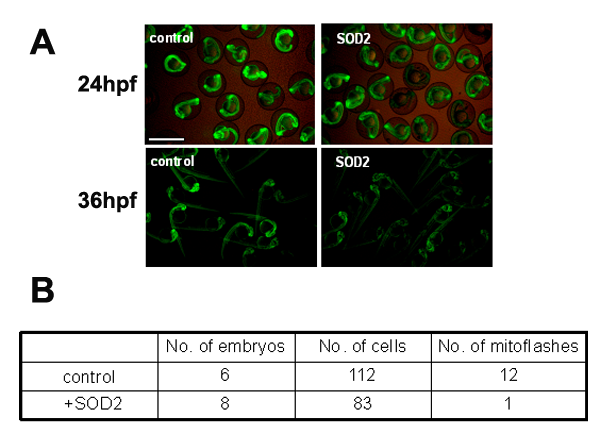
**

**S3 Fig.** **Basal cpYFP signal was decreased in zebrafish embryos overexpressing *sod2*.** Injection of sod2 mRNA decreased the cpYFP fluorescent level at 24 hpf and 36 hpf (Figure A), and suppressed mitoflash activity in red fibers of embryos at 2 dpf (Figure B). Tg(*β*-actin:mt-cpYFP) transgenic embryos were injected with *sod2* mRNA and were then used for measuring mitoflashes *in vivo*. The red and white fibers of the trunk skeletal muscles were recorded for mitoflashes, and imaged using a 40X, 1.3NA H_2_O immersion objective at a sampling rate of 1.57 s/frame on Zeiss 710 confocal microscope. Time-lapse images of 100 frames were acquired continuously each time. Scale bar, 10 µm.
