# Supplementary material for: Remodeling of Mitochondrial Flashes in Muscular Development and Dystrophy in Zebrafish
Source: PLoS One. 2015 Jul 17;10(7):e0132567. doi: 10.1371/journal.pone.0132567 (PMC4506073; doi:10.1371/journal.pone.0132567)
Supplement: S4 Fig — (DOC) [file pone.0132567.s004.doc]

**S4 Fig. Pearson correlation analysis of ΔF/F0 and T50 yielded coefficients of 0.4053 for S-type, -0.1488 for T-type, and 0.4039 for R-type mitoflashes.**
