# Supplementary material for: Remodeling of Mitochondrial Flashes in Muscular Development and Dystrophy in Zebrafish
Source: PLoS One. 2015 Jul 17;10(7):e0132567. doi: 10.1371/journal.pone.0132567 (PMC4506073; doi:10.1371/journal.pone.0132567)
Supplement: S5 Fig — (DOC) [file pone.0132567.s005.doc]

**
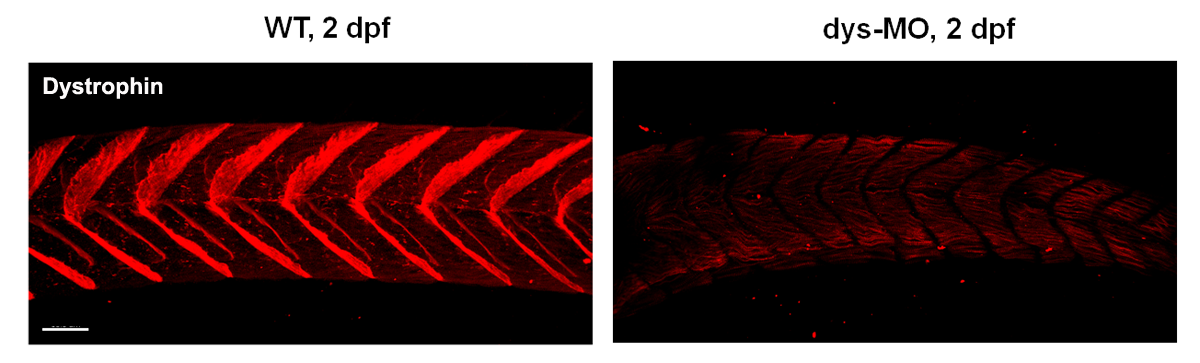
**

**S5 Fig. Dystrophin was efficiently reduced by dys-MO.** Dystrophin was expressed in V-shape in the neuromuscular junction in wild-type (WT) controls (n=32) but not in dys-MO injected embryos (n=46) at 2 dpf. This result is consistent with the previous report by Bassett et al. (2003). Scale bar, 50 µm.
