# Supplementary material for: Remodeling of Mitochondrial Flashes in Muscular Development and Dystrophy in Zebrafish
Source: PLoS One. 2015 Jul 17;10(7):e0132567. doi: 10.1371/journal.pone.0132567 (PMC4506073; doi:10.1371/journal.pone.0132567)
Supplement: S6 Fig — (DOC) [file pone.0132567.s006.doc]

**
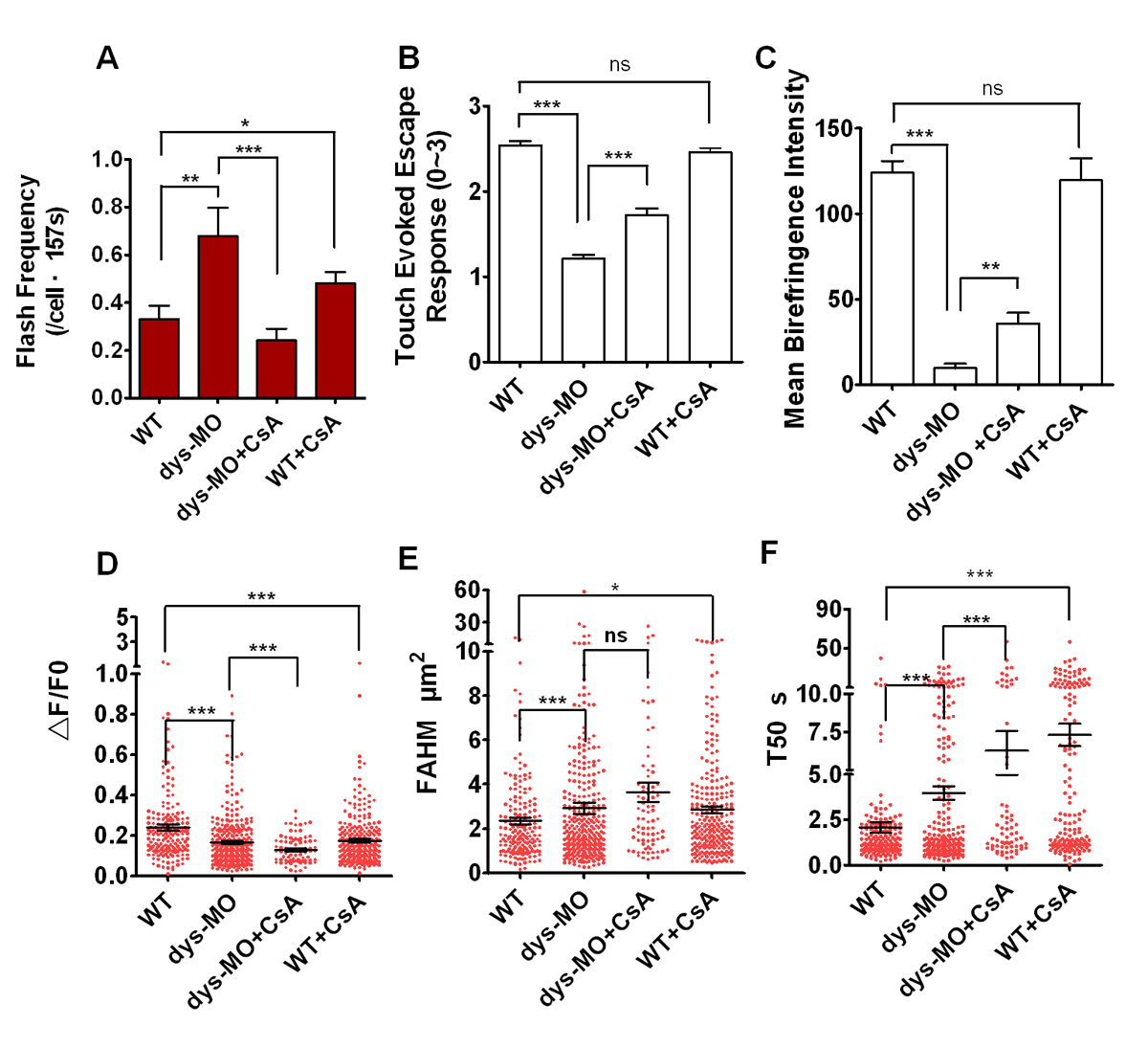
**

**S6 Fig. CsA partially rescued dysphophin morphant phenotype and decreased mitoflash frequency in red fibers of dystrophin morphants at 2 dpf.** (Figure A) The frequency of mitoflashes was increased in dys-MO morphants, which was reversed by CsA treatment (from 53 to 56hpf). (Figure B) Touch-evoked escape response was defective in dys-MO morphants at 56 hpf, which was partially rescued by CsA treatment (from 50 to 56 hpf). The touch response was not affected by CsA treatment in wild-type embryos. WT (n=137), dys-MO (n=315), dys-MO+CsA (n=114), and WT+CsA (n=134). (Figure C) The mean birefringence intensity was severely reduced in dys-MO morphants, which was partially restored by CsA treatment (from 53 to 56hpf). The mean birefringence was not affected by CsA treatment in wild-type embryos. WT (n=46), dys-MO (n=28), dys-MO+CsA (n=40), and WT+CsA (n=14). (D-F) ΔF/F0 (D), FAHM (E), and T50 (F) in WT, dys-MO, dys-MO+CsA and WT+CsA embryos at 56 hpf. (Figure D) ΔF/F0 was decreased in dys-MO morphants, which was further reduced after CsA treatment. Consistently, CsA treatment reduced ΔF/F0 amplitude in wild-type embryos. (Figure E) FAHM was increased in dys-MO morphants that could not be rescued by CsA treatment. FAHM was slightly increased by CsA treatment in wild-type embryos. (Figure F) The percentage of R-type mitoflashes was increased in dys-MO morphants that was partially rescued by CsA treatment. However R-type mitoflashes were also increased in wild-type embryos after CsA treatment. The percentages of S-type mitoflashes in WT, dys-MO, dys-MO+CsA, and WT+CsA embryos are 95.2%, 76.3%, 77.3% and 60.5%, respectively. The percentages of R-type mitoflashes in WT, dys-MO, dys-MO+CsA, and WT+CsA embryos are 3.0%, 11.7%, 22.7% and 26.3% respectively. Note that the mitoflash frequency was reduced in dys-MO morphants treated with CsA. Data were reported as median with interquartile range for (Figure D-F) and mean ± SEM for (Figure A-C). The nonparametric unpaired t test with Welch’s correction (Figure A-C) and Mann-Whitney test (Figure D-F) was applied to determine statistical significance of the differences as noted. *, *P* <0.05; **, *P* <0.01; ***, *P* <0.0001.
